# Association Between Cerebral Microbleeds and Depression in the General Elderly Population: A Meta-Analysis

**DOI:** 10.3389/fpsyt.2018.00094

**Published:** 2018-03-19

**Authors:** Ruiming Wang, Keqin Liu, Xiaoyun Ye, Shenqiang Yan

**Affiliations:** ^1^Department of Emergency, Hangzhou Hospital of Traditional Chinese Medicine, Hangzhou, China; ^2^Department of Neurology, Hangzhou First People’s Hospital, Hangzhou, China; ^3^Department of Nursing Education, School of Medicine, The Second Affiliated Hospital of Zhejiang University, Hangzhou, China; ^4^Department of Neurology, School of Medicine, The Second Affiliated Hospital of Zhejiang University, Hangzhou, China

**Keywords:** depression, prevalence, magnetic resonance imaging, microbleeds, meta-analysis

## Abstract

**Background:**

It remains unclear whether cerebral microbleeds (CMBs) are associated with depression in the general elderly population. We thus performed a meta-analysis to evaluate the relationship between depression and CMBs.

**Methods:**

A systematic literature search was conducted in EBSCO, PubMed, and Web of Science for relevant studies that assessed the relationship between depression and the prevalence of CMBs.

**Results:**

Five eligible studies including 7,328 patients were pooled in meta-analysis. The prevalence of CMBs was 18.0%. The prevalence of depression was 11.1%. The pooled analysis demonstrated odds ratio for CMBs and depression to be 1.187 (95% confidence interval 1.005–1.403; *p* = 0.043) with no evidence of statistical heterogeneity (*I*^2^ = 0.0%, *p* = 0.621).

**Conclusion:**

Our meta-analysis of available published data indicated an increased prevalence of depression in the subjects with pre-existing CMBs. This finding supports the vascular depression hypothesis. Further studies are needed to investigate the role of CMBs in the pathogenesis and progression of depression, which might provide a potential target for the prevention and treatment.

## Introduction

Depression is a common mood disorder in the elderly and is associated with high rates of morbidity and risk for mortality (1). The etiology of depression is incompletely understood, whereas evidence suggests a “vascular depression” hypothesis (2). Several studies have indicated that cerebral small vessel disease (CSVD) might play an important role in the etiologic factors of depression (3–5).

Cerebral microbleeds (CMBs), a subtype of radiological CSVD (6), are defined as small and rounded hypointense lesions on gradient recalled echo (GRE) or susceptibility weighted imaging (SWI) (7). Histopathological analysis indicated that CMBs consisted of hemosiderin accumulations from red blood cells that presumably had leaked out of small vessels (8), and early endothelial failure might be involved in its main pathological mechanism (9). A recent meta-analysis showed that higher levels of plasma endothelial biomarkers, including soluble intercellular adhesion molecule-1, soluble vascular cell adhesion molecule-1, e-selectin, and von Willebrand factor, were associated with higher odds of late-life depression (10). Although a brief analysis between CMBs and depression was performed in van Agtmaal’s study (10), the effect of race, imaging parameters, lesion location, and the coexisting leukoaraiosis on this relationship still remained unknown. Therefore, we performed a meta-analysis to determine whether an association between depression and CMBs exists, considering these potential confounds mentioned earlier, in the general elderly population.

## Materials and Methods

This meta-analysis is reported in accordance with the Preferred Reporting Items for Systematic Reviews and Meta-Analyses and Meta-analysis of Observational Studies in Epidemiology guidelines (11, 12).

### Search Strategy and Eligibility Criteria

We searched appropriate articles by systematic queries of PubMed, Web of Science, and EBSCO databases on 24 July 2017, using the following search terms: “micro(-)bleed(s)” or “micro(-)h(a)emorrhage(s)” in association with “depression” or “depressive” or “antidepressive” or “antidepressant(s)”. Two authors identified potentially relevant studies, resolving any uncertainties with a third author.

Both retrospective and prospective studies were eligible for inclusion if (1) the cohorts were at least above 45 years and free of dementia or major stroke, (2) had assessed depressive symptoms or depression in the cohort, and (3) had quantified the odds ratio (OR) in relation to the presence of CMBs on GRE or SWI. The exclusion criteria were listed as follows: (1) without detailed data; (2) the depressive symptoms or depression were evaluated after a stroke; (3) special cohorts, i.e., cohort of cerebral autosomal dominant arteriopathy with subcortical infarcts and leukoencephalopathy (CADASIL) patients; and (4) same cohort from an included study.

### Study Selection and Data Extraction

Two authors considered all titles and abstracts for eligibility in a systematic manner and went through all articles selected as relevant and extracted data independently. We extracted the following information on the study design: MRI parameters for CMBs detection, definition of depression, number and demographics of participants (including age, sex, and education level), mini mental state examination (MMSE) score of participants, number of participants with initial CMBs, number of participants with depression, and the characteristics of CMBs (burden and location) by using a unified data form.

### Data Analysis

A fixed-effects model (Mantel and Haenszel method) was used to calculate the pooled ORs and corresponding 95% confidence intervals (CIs), with weights calculated using the inverse variance method, because of the relatively small number of included studies and outcome events. Subgroup analysis was performed to isolate patients in the cross-sectional studies only. Sensitivity analyses were also performed based on the different MRI parameters or adjustments of potential confounds. Statistical heterogeneity was assessed using *I*^2^ statistics with inspection of the Forest plot. Publication bias was evaluated with Egger’s test, Begg’s test, and the Funnel plot. We repeated all analyses using random-effects models. All statistical analyses were performed with Stata 11.2 (StataCorp LP, College Station, TX, USA).

## Results

**Figure 1:**
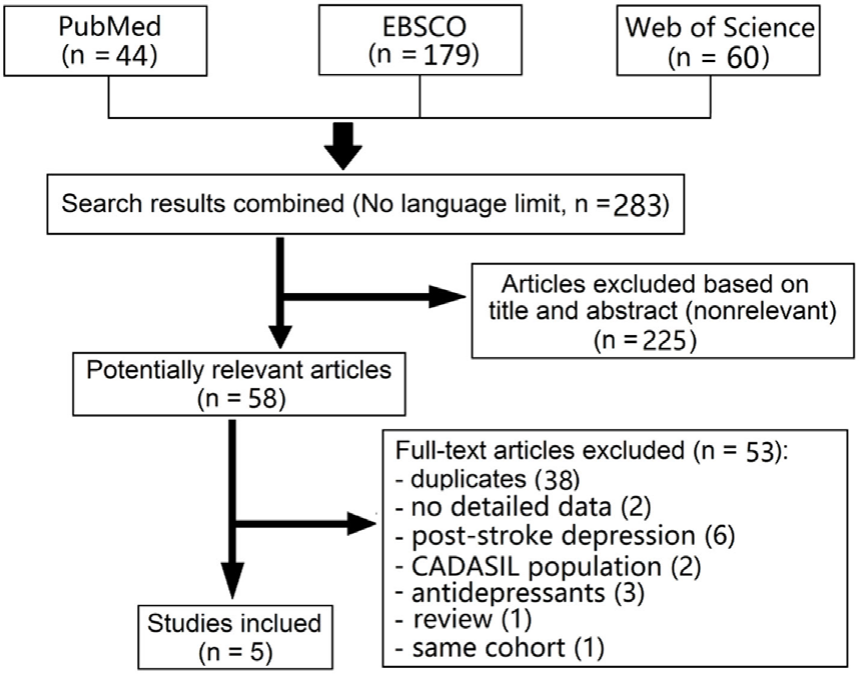
Flow diagram of literature search and study selection.

We identified 44 articles of PubMed, 179 of EBSCO, and 60 of Web of Science in our initial search. Six studies (all published) met our predetermined criteria; however, two studies were from a same cohort (13, 14). Finally, five studies were pooled in a meta-analysis (Figure 1) (3, 4, 13, 15, 16). Characteristics of the included studies are summarized in Table 1.

**Table 1:** Characteristics of the included studies.

Study reference	Design	Inclusion criteria	MRI parameters				Definition of depression
			Sequence	Field strength	Echo time	Slice thickness	
van Norden et al. (16); Western cohort	Prospective, the RUN DMC study	(1) Age between 50 and 85 years and (2) with cerebral SVD on neuroimaging	T2*-GRE	1.5 T	26 ms	6.0 mm	A score ≥ 16 on the CES-D Scale and/or the present use of antidepressive medication

Wu et al. (13); Asian cohort	Prospective, a case–control study	(1) 65 years old or older, with ability and will to give consent and (2) without physical disability	SWI	3.0 T	20 ms	1.5 mm	According to DSM-IV after a face-to-face interview

van Sloten et al. (3); Western cohort	Prospective, a longitudinal study, the AGES-Reykjavik study	(1) Free of dementia and (2) without baseline depressive symptoms	T2*-GRE, 3D	1.5 T	50 ms	3.0 mm	Geriatric Depression Scale cutoff score ≥ 6 at follow-up and/or new use of antidepressant medication

Direk et al. (4); Western cohort	Prospective, the Rotterdam study	(1) Aged 45 years or over and (2) were free of dementia	T2*-GRE, 3D	1.5 T	31 ms	1.6 mm	With a CES-D score of 16 or greater and clinical assessment

Xu et al. (15); Asian cohort	Prospective, a multiethnic study, the EDIS study	(1) Aged 60 years and above and (2) were screened with Abbreviated Mental Test and a self-report of forgetfulness	SWI	3.0 T	20 ms	1.5 mm	Part of the 12-item neuropsychiatric inventory

*The RUN DMC study, the Radboud University Nijmegen Diffusion tensor and Magnetic resonance Cohort study; SVD, small vessel disease; GRE, gradient-recalled echo; CES-D, the Center of Epidemiological Studies on Depression; SWI, susceptibility-weighted imaging; DSM-IV, diagnostic and statistical manual of mental disorders; the AGES-Reykjavik Study, the Age, Gene/Environment Susceptibility-Reykjavik study; the EDIS study, the Epidemiology of Dementia In Singapore study*.

Study demographics are summarized in Table 2. Collectively, these studies were composed of 7,328 patients with CMBs evaluation (study sample size range: 335–3,742), 1,319 (18.0%) of which had CMBs on initial MRI scans. Notably, patients received SWI (3.0 T) showed higher prevalence of CMBs (33.4–34.9%) than those received T2*-GRE (1.5 T) (10.4–17.3%) (3, 4, 13, 15, 16). About half of the patients with CMBs had exactly 1 CMB (15, 16). Depression or depressive symptoms occurred in 11.1% (range: 8.5–33.6%) of the entire population.

**Table 2:** Study demographics and outcomes.

Study	van Norden et al.	Wu et al.	van Sloten et al.	Direk et al.	Xu et al.	Total
Population size	500	335	1,949	3,799 (3,742)^a^	802	7,385 (7,328)
Age (years)	65.6 (mean)	72.5 (mean)	74.6 (mean)	58.7 (mean)	70.3 (mean)	65.2 (mean)
Male	284 (56.8%)	159 (47.5%)	846 (43.4%)	1,729 (45.5%)	369 (46.0%)	3,387 (45.9%)
None or primary education level	49 (9.8%)	–	367 (18.9%)	438 (11.5%)	520 (64.8%)	–
Microbleed prevalence	52 (10.4%)	112 (33.4%)	337 (17.3%)	538 (14.4%)	280 (34.9%)	1,319 (18.0%)
Baseline MMSE score	28.1 (mean)	24.9 (mean)	28 (median)	28.1 (mean)	23.6 (mean)	–
Depression	168 (33.6%)	65 (19.4%)	197 (10.1%)^b^	322 (8.5%)^c^	68 (8.5%)^d^	820 (11.1%)

*MMSE, mini mental state examination*.

*^a^Data available for 3,742 patients with microbleed evaluation*.

*^b^Follow-up data*.

*^c^Data were calculated from an odd ratio adjusted for age and gender*.

*^d^Data were extracted from the figure*.

**Figure 2:**
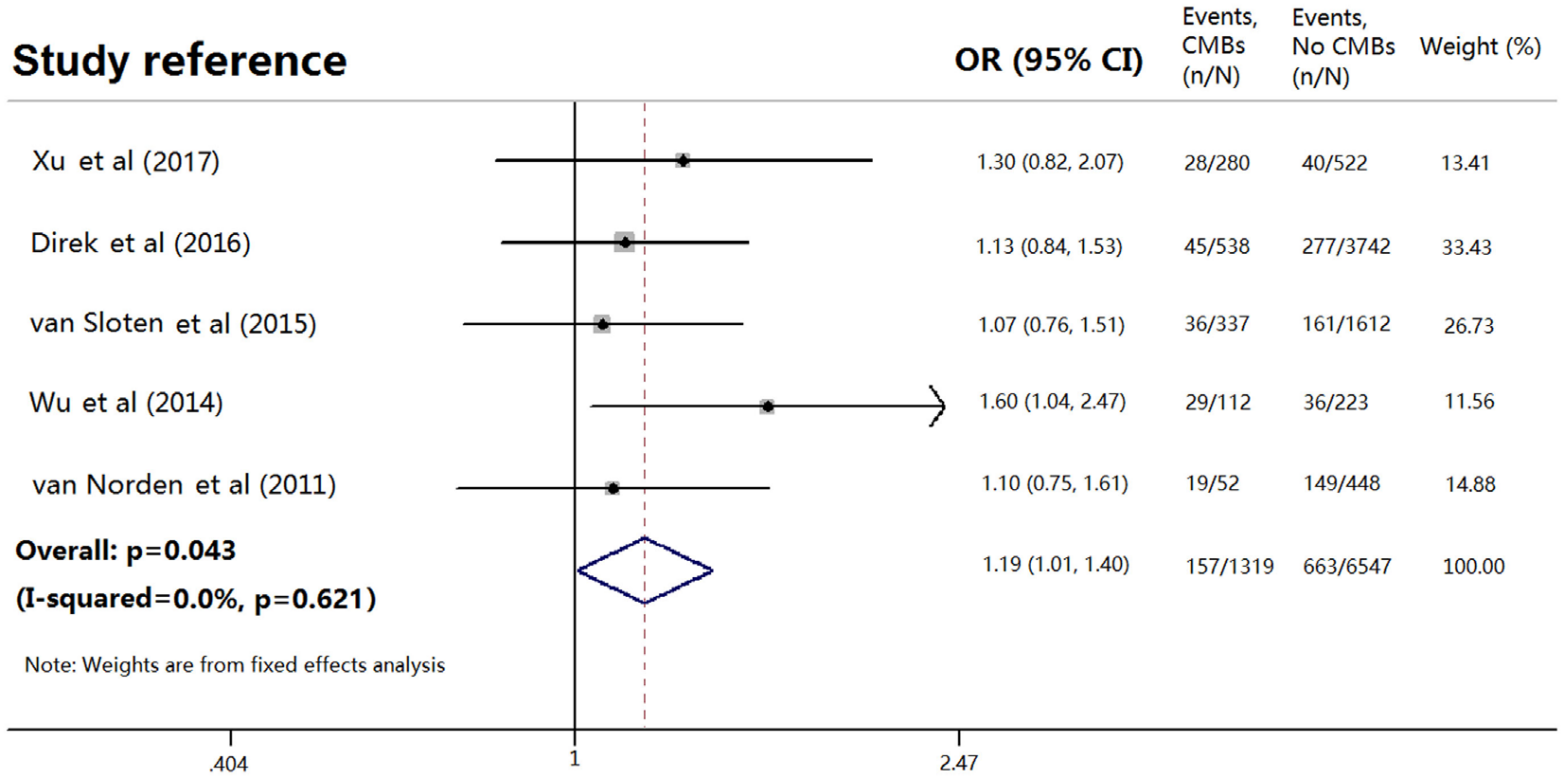
Meta-analysis of the association between depression and cerebral microbleeds (CMBs) in the general population. Adapted from Ref. (3, 4, 13, 15, 16).

**Figure 3:**
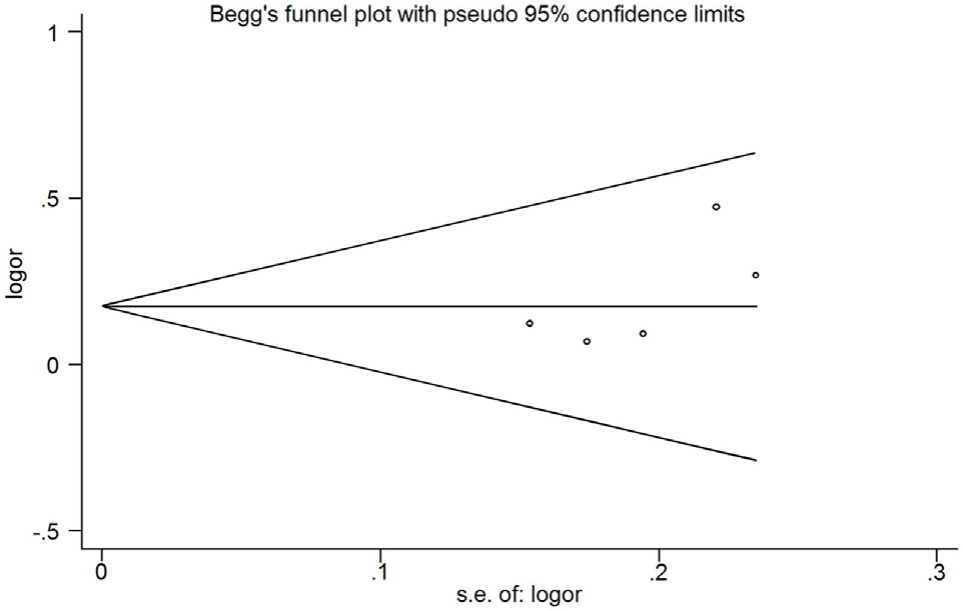
Publication bias from the studies about the association between depression and the presence of cerebral microbleeds.

Among patients with initial CMBs, 157 of 1,319 (11.9%) had depression compared with 663 of 6,009 patients (11.0%) without CMBs. Pooled analysis demonstrated OR for the presence of initial CMBs and depression to be 1.187 (95% CI 1.005 to 1.403; *p* = 0.043) with no evidence of statistical heterogeneity (*I*^2^ = 0.0%, *p* = 0.621) (Figure 2). There was no evidence of a publication bias either from the result of Egger’s test (*p* = 0.192) or Begg’s test (*p* = 0.462), and the shape of the funnel plot seemed symmetrical (Figure 3).

**Figure 4:**
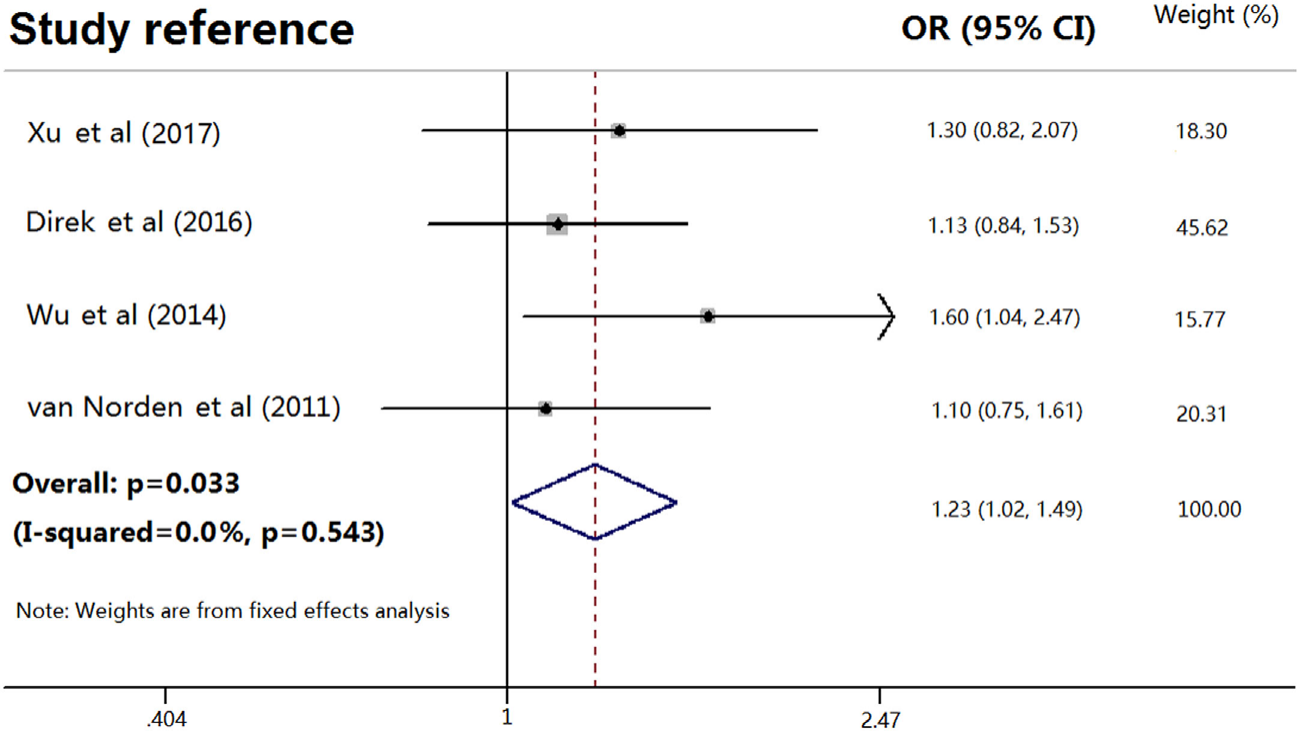
Meta-analysis of the association between depression and cerebral microbleeds in the cross-sectional studies only. Adapted from Ref. (4, 13, 15, 16).

One of the included studies is a longitudinal study with a 5.2-year follow-up (3). After excluding van Sloten et al’s study, pooled analysis of the rest four cross-sectional studies (4, 13, 15, 16), including 5,379 patients (982 with CMBs), demonstrated OR for the presence of CMBs and depression to be 1.230 (95% CI 1.017–1.489; *p* = 0.033) with no evidence of statistical heterogeneity (*I*^2^ = 0.0%, *p* = 0.543) (Figure 4). Pooled analysis of Asian cohorts that used 3.0 T MRI (13, 15), including 1,137 patients (392 with CMBs), demonstrated OR for the presence of CMBs and depression to be 1.443 (95% CI 1.052–1.980; *p* = 0.023). However, no significant association was found in the Western cohorts that used 1.5 T MRI (OR = 1.102, 95% CI 0.906–1.342; *p* = 0.332) (3, 4, 16). Based on the limited data (4, 11), lobar CMBs seemed to be associated with depression (OR = 1.636, 95% CI 1.107–2.416; *p* = 0.013). After adjusting for some potential confounds, such as age, the severity of leukoaraiosis, MMSE, and so on, the presence of CMBs was still associated with depression (OR = 1.279, 95% CI 1.008–1.623; *p* = 0.043) (3, 4, 13). All analyses were consistent using a random-effects model.

## Discussion

Our meta-analysis in more than 7,000 subjects with MRI evaluation reported a positive relationship to exist between CMBs and depression. Asian population, higher magnetic field strength, or lobar location of CMBs might strengthen this relationship, even after adjusting for some potential confounds, such as age and the severity of leukoaraiosis, in a minority of studies. These findings support the vascular depression hypothesis.

The prevalence of CMBs was reported from 4.7 to 15.3% in normal individuals (17–20). Interestingly, the two Asian cohorts had a higher proportion of patients with at least one CMB (33.4–34.9%) (13, 15), than the three Western cohorts (10.4–17.3%) (3, 4, 16), which might be explained by the race-ethnicity difference. On the other hand, the difference in MRI parameters among each study might also affect the prevalence of CMBs. It has been demonstrated that increased field strength, longer echo time, and higher spatial resolution (3D Fourier transform technique) can increase the sensitivity of CMBs detection (7). Susceptibility effect increased with higher field strength and blooming effects are therefore predicted to be greater, thus a higher field strength (i.e., 3.0 T versus 1.5 T) appears to improve CMB conspicuity (7). CMBs could be missed due to a large slice thickness and/or a large interslice gap of MRI scans (21).

Several studies have demonstrated the relationship between CSVD and depression (3–5). Both leukoaraiosis and CMBs were proven to be associated with depression in van Agtmaal et al’s meta-analyses (10). However, only four studies were enrolled to investigate the relationship between CMBs and depression in the meta-analyses (3, 4, 13, 14). More seriously, the included studies of Wu et al and Feng et al were from the same cohort (13, 14). In addition, leukoaraiosis is highly correlated with CMBs (22). It is thus unclear if the presence of CMBs is the independent predictor or rather severity of CSVD overall. In a special population of CADASIL, Park et al demonstrated that leukoaraiosis, not CMBs, is closely associated with depression (23), while Noh et al found depressive emotional disturbances was associated with multiple (≥10) CMBs, but not leukoaraiosis (24). Only a few studies adjusted the severity of leukoaraiosis in multivariate regression analyses (4, 13, 15). On the other hand, the influence of antidepressant use was not taken into account, as most previous studies were cross-sectional. In the population-based Rotterdam Study, the use of serotonin reuptake-inhibiting antidepressants was not related to the presence of CMBs (25); however, the use of antidepressant was proven to be associated with a higher CMBs incidence in the individuals without CMBs at baseline (26). Therefore, the severity of concomitant leukoaraiosis and previous use of antidepressants should be taken into consideration in future investigations.

The underlying mechanisms of pathological association between CMBs and depression are still unknown, which might be explained by several pathways. First, histopathologic analysis of CMBs generally found that these lesions indicated widespread damage of surrounding gliosis or even frank necrosis or infarction, resulting in microstructural damage of the surrounding white matter (8). In this way, CMBs could cause structural disruptions of white matter tracts that are relevant for mood regulation and predispose the individual to the development of depression. Second, microvascular dysfunction in the subjects with CMBs is closely linked to and interrelated with chronic low-grade inflammation and/or oxidative stress (27), while inflammation has been linked to depression or post-stroke depression via perfusion deficits or increased expression of serotonin receptor (28, 29). Third, lobar CMBs were found to be associated with depression, supporting the pathology of amyloid angiopathy, which played an important role in the pathophysiology of Alzheimer’s disease (30). Meanwhile, late-life depressive symptoms represent an early manifestation of dementia (30). Fourth, antidepressant use might damage the endothelial layers, lead to activation of hemostatic mechanisms, inhibit the reuptake of serotonin by platelets from the blood, reduce intracellular serotonin concentrations, thereby decrease platelet aggregation and increase the risk of CMBs (26).

There were several limitations of our study. First, this meta-analysis had inherent biases associated with the use of observational studies, and most of them were cross-sectional studies. All studies were subject to selection bias since not every individual underwent MRI. Second, the results might be affected by confounding variables, since some of the studies did not provide full information of baseline characteristics likely to be associated with CMBs. Despite the most important influencing factors of age and leukoaraiosis, previous history of depression, personality disorders, and the psychiatric comorbidities (including substance abuse) are also major problems in cross-sectional analyses. Future studies of large sample size or individual data meta-analyses that contain all of these potential confounds are needed to clarify this issue. Third, the MRI parameters used varied among included studies. Echo time, field strength, slice thickness, and interslice gap can affect the sensitivity of CMBs detection. Fourth, there might be a possible bias, since only one study detected a positive relation between any CMBs and depression (13); however, the weight of this included study was acceptable (11.56%). Fifth, the relationship between depression and the number of CMBs remains unclear based on recent data. It is possible that the association would be much stronger for higher number of CMBs, further studies are thus needed.

In conclusion, our analysis showed that the presence of CMBs might be associated with depression in the general population, which was in agreement with the vascular depression hypothesis. Future large multicenter studies and individual patient data meta-analysis are needed to demonstrate the role of CMBs in the pathogenesis and longitudinal progression of depression in the general elderly population.

## Author Contributions

SY: design of the work; interpretation of data for the work; revising the work for important intellectual content; and final approval of the version to be published. RW: acquisition and analysis of data for the work; drafting the work; revising the work for important intellectual content; and interpretation of data for the work. KL: acquisition and analysis of data for the work; drafting the work; revising the work for important intellectual content; and interpretation of data for the work. XY: acquisition and analysis of data for the work; drafting the work; and revising the work for important intellectual content.

## Conflict of Interest Statement

The authors declare that the research was conducted in the absence of any commercial or financial relationships that could be construed as a potential conflict of interest. The reviewer LV and handling editor declared their shared affiliation.
